# Hybrid Sol-Gel Surface-Enhanced Raman Sensor for Xylene Detection in Solution

**DOI:** 10.3390/s21237912

**Published:** 2021-11-27

**Authors:** Verena Weber, Laura Brigo, Giovanna Brusatin, Giovanni Mattei, Danilo Pedron, Roberto Pilot, Raffaella Signorini

**Affiliations:** 1 Department of Chemical Science, University of Padua, Via Marzolo 1, I-35131 Padova, Italy; verena.weber@unipd.it (V.W.); danilo.pedron@unipd.it (D.P.); roberto.pilot@unipd.it (R.P.); 2 Department of Industrial Engineering, University of Padua, Via Marzolo 9, I-35131 Padova, Italy; laurabrigo@gmail.com (L.B.); giovanna.brusatin@unipd.it (G.B.); 3 Department of Physics and Astronomy “Galileo Galilei”, University of Padua, Via Marzolo 8, I-35131 Padova, Italy; giovanni.mattei@unipd.it; 4 Consorzio INSTM, Via G. Giusti 12, I-50121 Firenze, Italy

**Keywords:** xylene, SERS sensor, nanostructures, sol-gel film

## Abstract

This paper reports on the fabrication and characterization of a plasmonic/sol-gel sensor for the detection of aromatic molecules. The sol-gel film was engineered using polysilsesquioxanes groups to capture the analyte, through π-π interaction, and to concentrate it close to the plasmonic surface, where Raman amplification occurs. Xylene was chosen as an analyte to test the sensor. It belongs to the general class of volatile organic compounds and can be found in water or in the atmosphere as pollutants released from a variety of processes; its detection with SERS is typically challenging, due to its low affinity toward metallic surfaces. The identification of xylene was verified in comparison with that of other aromatic molecules, such as benzene and toluene. Investigations were carried out on solutions of xylene in cyclohexane, using concentrations in the range from 0 to 800 mM, to evaluate the limit of detection (LOD) of about 40 mM.

## 1. Introduction

Surface enhanced Raman scattering (SERS) has been attracting much attention in the scientific community, especially in the field of chemical sensing, owing to its intrinsic high sensitivity and molecular specificity [1,2]. It has been successfully applied in different fields for the detection of materials such as explosives [3,4], toxic industrial chemicals [5], food contaminants and preservatives [6,7], biomolecules [8], bacteria [9], and dyes in works of art [10].

Volatile organic compounds (VOCs), in particular benzene, toluene and xylene (BTX), are a class of pollutants that require close monitoring due to their well-documented toxicity and widespread diffusion in the environment [11]. BTX are commonly used in the chemical industry for the production of many substances such as plastics, fibers, detergents, pesticides, paints, etc. [12]. BTX can contaminate water and soil as a consequence of accidental or fraudulent release of industrial waste, oil and gas extraction, leakage from underground storage tanks and so on [13]; importantly, they are also responsible for air pollution, as they can be released from combustion engines, industrial processes, and solvent use [14].

Ultra-sensitive and selective detection of toxic VOCs in traces is essential for environmental sustainability and human health. The measurement of VOCs, together with their identification, in the gaseous or liquid phase, is a challenging task that has been exploited with many different techniques. As an example, packed column gas chromatography has been combined with NMR techniques for the estimation of benzene and heavier aromatics in commercial gasoline [15]; solid-phase microextraction technique, followed by gas chromatography-mass spectrometry separation and detection, has been used for the determination of polycyclic aromatic hydrocarbons and benzene, toluene, ethylbenzene and xylene in snow water and water samples [16,17]. In addition, new methods, one based on the extraction solvent and combined with liquid chromatography [18], the second based on solid phase microextraction and combined with ultraviolet absorption spectroscopy, were employed to detect aromatic hydrocarbons in water [19]. All of these methods have high sensitivity and selectivity, but are costly and cannot be tailored for real-time analysis in the field. Only recently a development of a hand-portable system based on liquid chromatography incorporating a spectrally wide absorption detector was reported for the detection of polycyclic aromatic hydrocarbon mixtures [20]. A different approach is based on the detection of organic vapors through the observation of the Localized Surface Plasmon Resonance (LSPR) band of different nanostructures [21,22,23]. These sensors reveal the presence of organic compounds due to the variation of absorbance intensity, wavelength position, or bandwidth in the UV-VIS absorption spectrum of the plasmonic nanostructure. Although these works reported low detection limits (LODs) and high selectivity, the drawback of exploiting the LSPR band remains the indirect detection of the analyte: the variation of the plasmonic property is observed and is not directly a property of the analyte species. To overcome this drawback, SERS could be an interesting alternative detection technique. Only a few publications in the literature reported SERS experiments for the detection of organic compounds and the detection of VOCs [24,25,26,27,28,29,30,31].

In order to improve the selectivity and/or sensibility in SERS detection experiment, the affinity of the analytes to the substrate is often improved in several ways: for example, by functionalizing their surface with suitable receptors, by using aptamers or molecular imprint polymers [32], diazonium salt [33], polydimethylsiloxane (PDMS) [27], hydrophobic sponges [28], and metal-organic frameworks (MOFs) [34]. In addition, hybrid organic-inorganic sol-gel films have been proposed for separation and sensing applications [35,36]. In particular, bridged polysilsesquioxanes are an interesting class of versatile functional materials: by changing the bridged compound, one can exploit many different functional groups, ranging from disulfides to aromatics and ureic groups, showing affinity to different analytes. These polysilsesquioxanes have been exploited as VOC adsorbents [28,37]. The use of polysilsesquioxanes for the detection of VOC has also been tested, through LSPR in coated substrates [38]. Moreover, the use of hybrid sol-gel materials allows for fine-tuning of the porosity of the final films [39].

In this work, a new plasmonic/sol-gel sensor was developed for the selective detection of xylene. A novel SERS sensing strategy is here proposed; it is based on the interaction of VOC molecules with a hybrid organic/inorganic sol-gel film, in order to avoid time-consuming extraction techniques and obtain a high selectivity. The active structure is prepared by assembling core-shell (silica-gold) nanoparticles (NPs) on a glass substrate, functionalized with a silane molecule. On top of the SERS substrate, a thin hybrid organic-inorganic sol-gel layer is spin-coated: the sol-gel coating is synthesized from a precursor bearing a diphenyl moiety, which is expected to capture aromatic compounds through π-π interactions. The idea of coupling the sol-gel with the plasmonic substrate opens the way to a new class of sensors, whose affinity toward different analytes can be suitably tuned by properly choosing the characteristics of the sol-gel precursors. The plasmonic/sol-gel substrate is simple, easy to realize, and very versatile. It is demonstrated that it allows one to clearly distinguish xylene from other molecules of small aromatics, like benzene, with a promising detection limit (LOD), measured in cyclohexane solutions. The obtained results, in terms of LOD, are not yet competitive with other reported results in the literature, but leave the way open to further developments and exploitation. 

## 2. Materials and Methods 

### 2.1. Chemicals

Gold (III) chloride solution 30% w/w in dilute HCl (CAS 16903-35-8), silver nitrate (CAS 7761-88-8), silver acetate (CAS 563-63-3), sodium citrate dihydrate (CAS 6132-04-3), Tetraethyl orthosilicate (TEOS) (CAS 78-10-4), Tetrakis(hydroxymethyl)phosphonium 80% in water (THPC) (CAS 124-64-1), (3-Aminopropyl)trimethoxysilane (APTMS) (CAS 13822-56-5), Tetraoctylammonium bromide (CAS 14866-33-2), sodium borohydride (CAS 16940-66-2), formaldehyde solution (CAS 50-00-0), potassium carbonate (CAS 584-08-7), 1-Dodecanethiol (CAS 112-55-0) and Polyvinylpyrrolidone (average mol wt 40,000) (CAS 9003-39-8), thiophenol (or benzenethiol) (CAS 108-98-5), para-xylene (CAS 106-42-3), purchased from Sigma-Aldrich, m-PEG-SH (mol wt 2000) (CAS 134874-49-0), Laysan Bio Inc., and bis(triethoxysilyl)biphenyl polysilsesquioxanes, (CAS 123640-93-7), H_2_SO_4_ (CAS 7664-93-9), NH_4_OH (CAS 1336-21-6), H2O2 (CAS 7722-84-1) purchased from Sigma-Aldrich (96%), are used without further purification. Soda lime cover slip glasses were purchased from Thermo Scientific. The synthesis was carried out in Milli-Q deionized filtered water or in toluene (CAS 108-88-3).

### 2.2. Synthesis of Materials

#### 2.2.1. Synthesis of Nanoparticles and Nanoshells

Au NPs in aqueous solution were synthesized using the citrate Turkevich method [40]: 1 mL of a 39 mM aqueous solution of sodium citrate was added to 9 mL of a 1 mM solution of gold (III) chloride 30% w/w in dilute HCl, under reflux conditions, at 25 °C. The reaction was kept for 30 min under vigorous stirring.

Gold nanoshells (NSs) were prepared with a silica core and a gold shell [41]. The silica particles were synthesized using the Stöber method [42], based on sol-gel chemistry. An amount of 2 mL of 30% ammonium hydroxide solution was added to 50 mL of ethanol, under vigorous stirring, and then 1.5 mL of TEOS was slowly and dropwise added. The reaction mixture was kept overnight under stirring. For the functionalization of silica particles with APTMS, 10 µl of APTMS was added to 10 mL of NPs in ethanol and the solution was kept overnight stirring and heating at 80 °C. The solution (SiO_2_@APTMS) was then purified by centrifugation and dispersed in ethanol. Small gold NPs for silica-APTMS functionalization were synthesized by adding to 4.5 mL milli-Q water, 50 µl of a 1M NaOH solution, and subsequently 0.1 mL of a 68 mM THPC solution. The mixture was kept under vigorous stirring for 5 min and then 0.2 mL of a 1% aqueous HAuCl_4_ solution was quickly added. The final NS samples were prepared by adding 150 μL of SiO_2_@APTMS@Au to 8 mL of HAuCl_4_ solution and finally adding 50 μL of formaldehyde.

#### 2.2.2. Fabrication of SERS Substrates

**Figure 1:**
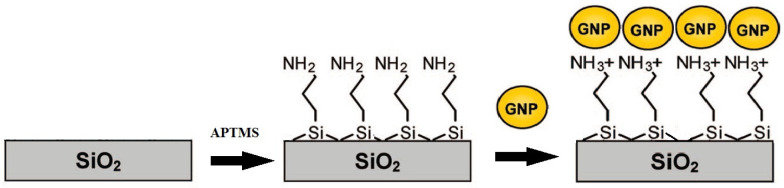
Scheme of the gold nanostructure deposition onto glass.

Plasmonic substrates were prepared according to the scheme shown in Figure 1 [43,44].

The glass slides were quickly cleaned with acetone, distilled water and finally with an acid piranha solution (H_2_SO_3_:H_2_O_2_ 3:1) at 80 °C for 1 h. The slides were then rinsed in distilled water and cleaned one more time with basic piranha (NH_4_OH:H_2_O_2_ 5:1) at 80 °C for 20 min. Their functionalization was accomplished by immersion in a 1% APTMS solution in toluene at 60 °C. The dried glasses were then immersed into a proper dilute colloidal nanostructure solution and kept at room temperature for 1 night, to allow the nanostructures to deposit onto the glass. The SERS substrates were finally cleaned with water to eliminate excess nanostructures on the substrate and stored in an inert environment.

#### 2.2.3. Synthesis of the Sol-Gel Matrix

**Figure 2:**
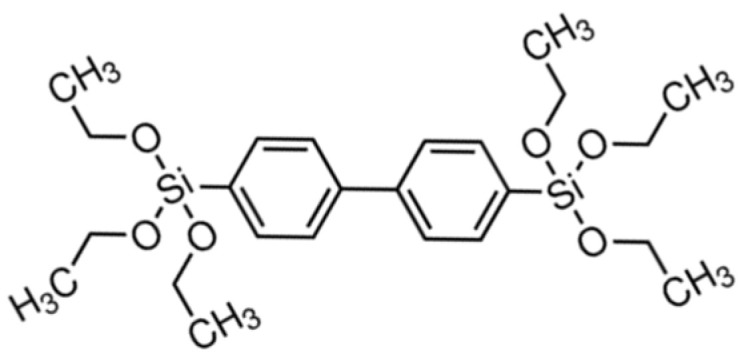
Molecular structure of bis(triethoxysilyl)biphenyl polysilsesquioxanes (TEPS), a precursor in sol-gel synthesis.

The precursor used for the synthesis of the hybrid film was bis(triethoxysilyl)biphenyl polysilsesquioxanes (TEPS), whose molecular structure is depicted in Figure 2. 

A solution of the sol−gel precursor, ethanol (EtOH) and bidistilled water was mixed at room temperature in a 1/6 (molar ratio) precursor/H_2_O mixture, using 1 N hydrochloric acid (HCl) as the catalyst. The organic moiety in the hybrid network is specifically chosen for exhibiting a high-affinity interaction with aromatic hydrocarbons. Aromatic analyte molecules, penetrating into the porous sol-gel film, interact with the phenyl bridging groups of the organic unit by π-π stacking interaction [45,46]. The use of these bridged silsesquioxane sol-gel precursors results in the realization of a film characterized by a pore volume fraction of 35% [47], allowing the diffusion of small molecules.

The basic sol-gel synthesis is performed in the presence of HCl as an acidic catalyst and water in a 15 to 40 g/L dilute sol solution of the precursor, depending on the desired final film thickness. The solution was then spin coated on a soda lime glass (about 2000–6000 rpm for 30–60 s) and subjected to a heat treatment of about 30 min at 60 °C. Through this method, already reported in the literature [38], films of thicknesses ranging from 50 to 300 nm could be obtained simply by varying the concentration of precursor solution and the spin coating parameters.

### 2.3. Characterization Techniques

#### 2.3.1. UV-Vis Absorption Spectroscopy, Dynamic Light Scattering and Z-Potential Measurements

UV-Vis absorption spectroscopy spectra were collected with a Cary5 (Varian) spectrophotometer. All measurements were made in quartz cells with an optical path of 1 or 10 mm. Dynamic Light Scattering (DLS) and Z-potential measurements were performed on NPs in solution with a Malvern Zetasizer Nano ZS with a 633 nm laser excitation. 

#### 2.3.2. Raman

Raman and SERS measurements were performed using a home-made Micro-Raman setup. A He–Ne laser, working at 633 nm (Melles Griot, output power 35 mW), was used as an excitation light source. The laser beam was coupled to a microscope (Olympus BX 40) and focused on the sample using a 50× and a 20× objective (Olympus SLMPL, NA 0.75 and 0.4, respectively) for solid and liquid samples, respectively. The backscattered Raman signal was separated from the Rayleigh scattering by an edge filter and analyzed with a 320 mm focal length imaging spectrograph (TRIAX-320 ISA) and a liquid nitrogen-cooled CCD camera (Spectrum One, JobinYvon). The typical spot diameter at the focus was between 1 and 3 µm. The laser power on the samples ranged from 24 to 0.12 mW. 

#### 2.3.3. Atomic Force Microscopy

The instrumentation used was an NT-MDT (NT- MDT-Europe B.V., Nuenen, the Netherlands) system, working in air with a piezoelectric scanner at 3 µm. The AFM images were analyzed with appropriate software, giving the NP dimensions. Measurements were made in tapping mode with a lock-in system to increase the signal-to-noise ratio.

#### 2.3.4. Transmission Electron Microscopy

Samples were prepared by putting some drops of NP solution on a copper grid. To prevent charge build-up at the sample surface, we coated the samples with a thin layer of conductive carbon material. The instrumentation used was a Field-Emission Gun (FEG) Tecnai F20 Super-twin (S)TEM (transmission electron microscopy) operating at 200 keV and equipped with an EDAX (energy-dispersive X-ray spectrometer), a Gatan EELS (Electron energy loss spectrometer), and a scanning TEM attachment.

## 3. Results and Discussion

### 3.1. Optical and Morphological Characterization of Nanoparticles, Nanoshells, and Substrates

**Figure 3:**
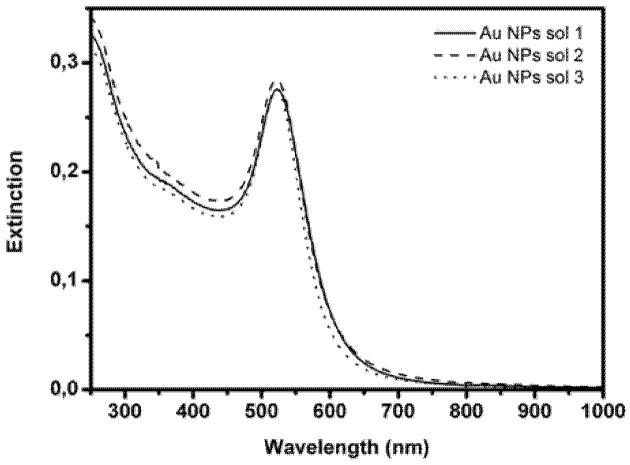
Extinction spectra of three different colloidal solutions of Au NPs.

The synthesis of Au NPs is very reproducible, as demonstrated by the UV-Vis spectra of three different solutions, reported in Figure 3, showing good overlap. The stability of the Au NP solutions was also tested through Z-potential measurements: values of about −40/−45 mV were obtained, confirming the high stability of these NPs in solution.

By fitting the UV-Vis spectrum in solution, with the Mie-Gans relation, and extracting the extinction cross section [48], the dimension and concentration of Au NPs were estimated. The resulting diameter and concentration values are comparable to those derived from TEM measurements.

**Figure 4:**
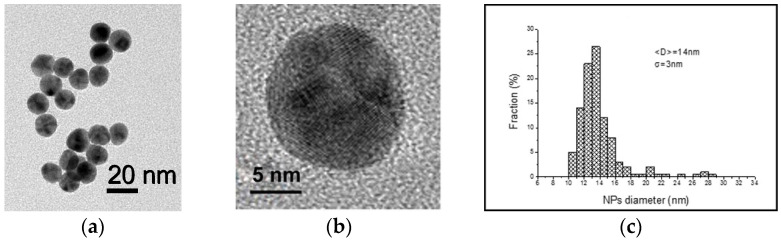
TEM image of a gold colloidal solution (**a**) 20 nm scale bar, with the zoom of a single NP (**b**), 5 nm scale bar, and histogram of the dimensional distribution (**c**).

A first measurement of the particle radius was performed through dynamic light scattering, giving a hydrodynamic radius of R1(DLS) = (58 ± 8) nm. The radius of the TEM analysis returned a smaller radius of R1(TEM) = (46 ± 3) nm, as can be observed in Figure 4. The final concentration of NPs, calculated from the average diameter extracted by TEM analysis (14 nm), is approximately 7∙10^12^ NPs/mL.

**Figure 5:**
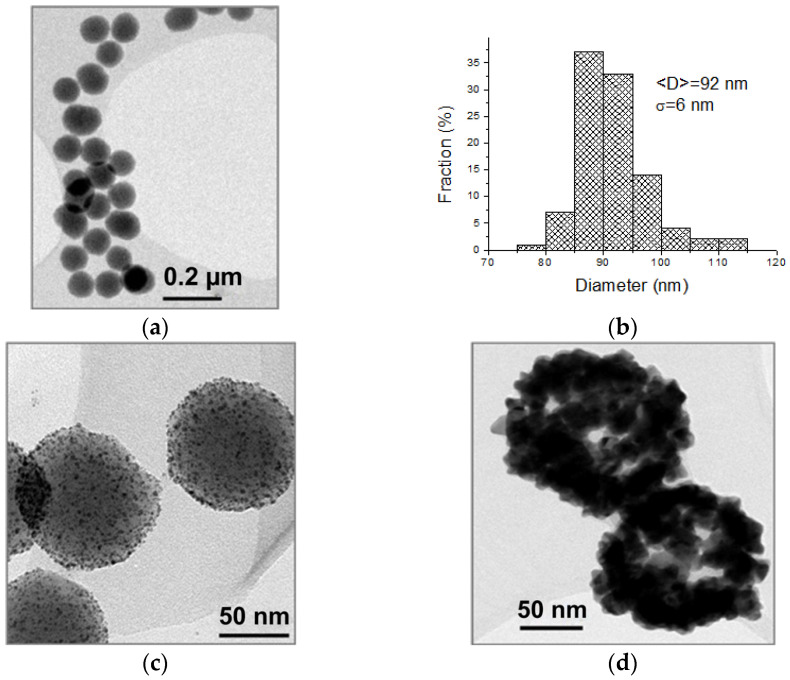
TEM analysis of bare SiO2 core NPs (R1 = 46) NPs (**a**) and histogram of the dimensional distribution (**b**). TEM images of SiO2@APTMS@Au (**c**) and Au NSs (R1 = 46; R2 = 65) (**d**).

The silica cores, used for the synthesis of NSs, present a very sharp distribution with a standard deviation of only 6.5% of the average radius, as observed from the TEM image in Figure 5a and its histogram distribution (b). EDX measurements on bare silica particles give 67 % atomic presence of oxygen and 33% of silica, as expected. Using the mean radius value of the TEM analysis, the concentration of the native silica NP solution was 1 × 10^13^ NPs/mL.

The solution of small Au NPs obtained had a concentration of 4∙10^14^ NPs/mL, calculated by estimating the radius of NPs 1 nm. The homogeneous decoration of the silica cores with gold nuclei is visible in the TEM image of Figure 5c. 

From the difference between the measured NS radius and the SiO2@APTMS radius, the final shell thickness is calculated (R2). From the average TEM radius and the effective average shell thickness, the internal, R1, and the external, R2, NS radii were obtained (see Figure 5c and Table 1). Figure 5d shows that the outer shell is partly incomplete.

**Figure 6:**
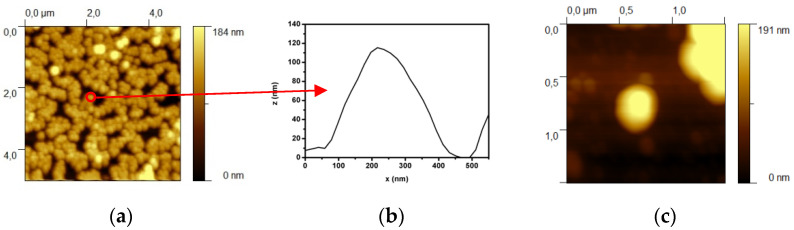
AFM images of the SiO_2_(R1 = 46)@APTMS sample (**a**) and Au NSs (R1= 46; R2= 67) (**c**) and example of a single NP profile (**b**). The red arrow and the open circle indicate the particle chosen for profile extraction.

The SiO_2_@APTMS particles were also analyzed by AFM; Figure 6a shows a high particle density, where the particles tend to aggregate in a hexagonal closed-packet conformation. However, particle diameters could be measured through appropriate software by extracting the nanoparticle profile, shown, as an example, in Figure 6a. From a statistical measurement of 10 profiles extracted from the AFM image, a particle dimension of (122 ± 11) nm was calculated.

The dimension estimated through AFM measurements is larger than the dimension measured through TEM. The reason is principally that the AFM images are influenced by the convolution of the AFM tip. For the NS sample, 10 profiles are extracted and the average diameter with its standard deviation is calculated. From the difference between the measured NS diameter and the SiO2@APTMS diameter, the shell thickness is calculated. 

**Figure 7:**
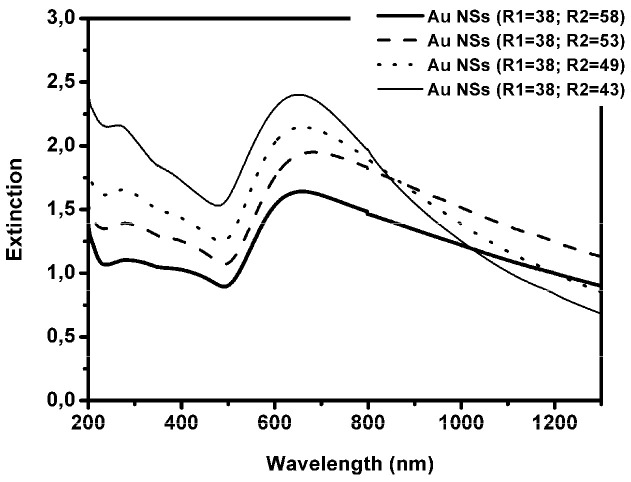
UV-Vis-NIR spectra of four different Au NSs (R1 = 38; R2 = x) samples.

The UV-Vis-NIR spectra of different NS samples, obtained by adding different amounts of SiO2 (R1 = 38)@APTMS@Au to the aged gold precursor solution, are shown in Figure 7. 

In Au NSs (R1 = 46; R2 = y) samples a band, centered between 600 and 700 nm, is present. This reflects the presence of an incomplete core-shell system [49] visible also in the TEM images in Figure 5d.

**Figure 8:**
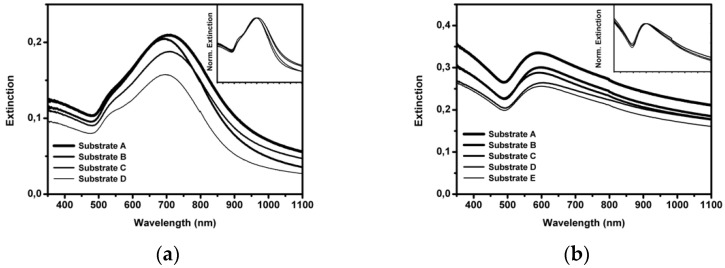
Extinction spectra of different Au NP substrates (**a**) and Au NS (R1 = 38; R2 = 49) substrates (R1 = 38; R2 = 49) (**b**); normalized spectra in the insets.

Both Au NPs and Au NSs were used to prepare substrates and exploit them for SERS sensing applications. The reproducibility of the plasmonic substrates was tested by preparing samples in the same experimental conditions and by checking their plasmonic resonance spectral position through UV-Vis spectroscopy. In Figure 8 the UV-Vis-NIR spectra of Au NPs and Au NSs (R1 = 38; R2 = 49) substrates are shown.

The plasmonic extinction band of colloidal Au NPs was modified after deposition on a solid support. This is due to the formation of aggregates on the glass slide involving the presence of NP hot spots. 

**Figure 9:**
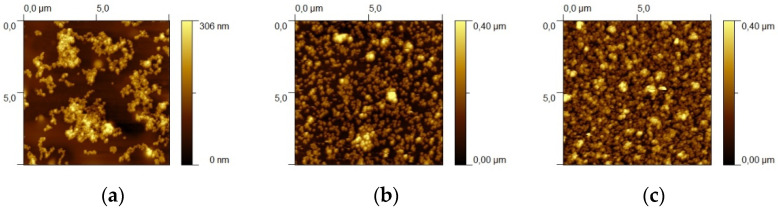
AFM images of Au NSs (R1 = 46; R2 = 69) substrates (R1 = 46; R2 = 69) with a coverage degree of about 20% (**a**), 50% (**b**) and 70% (**c**).

In the case of Au NS substrates, the realization of substrates at varying NS concentrations was tested through AFM. The AFM images on Au NS (R1 = 46; R2 = 69) substrates, realized with three different Au NS concentrations, are shown in Figure 9.

From the AFM images, it can be observed that at higher coverage degrees the substrates appear more homogeneous. Therefore, the conditions for obtaining coverage degrees of about 70–75% were used for substrate realization.

**Figure 10:**
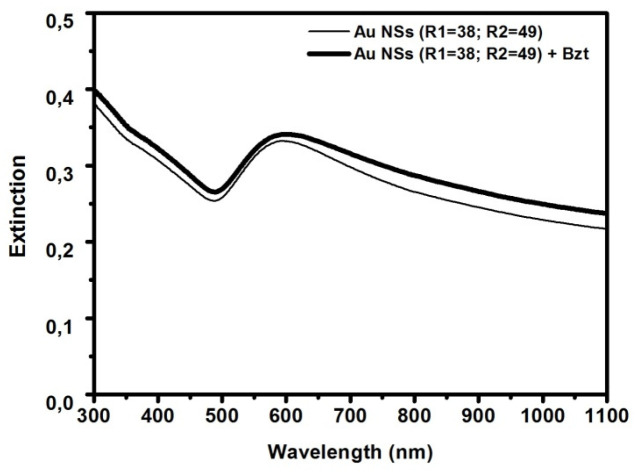
Extinction spectrum of Au NSs (R1 = 38; R2 = 49) before and after functionalization with benzenethiol.

The stability in the presence of different environments was checked by optical characterization in a phosphate buffer solution and in neutral and acidic methanol. The final stability test was performed after functionalization of the substrate with the analyte of interest. In Figure 10, a stability test is shown in a 1 mM benzenethiol solution in methanol on Au NSs (R1 = 38; R2 = 49).

As can be observed, the Au NS substrate is stable and does not undergo spectral modifications when immerged into a methanol solution. 

**Figure 11:**
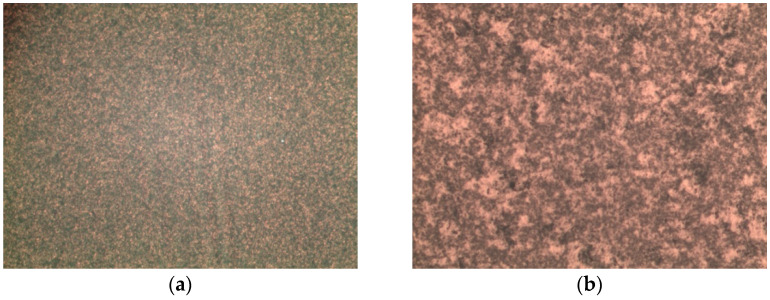
Microscopy images, collected with a 50× objective on the micro-Raman setup, of Au NSs (**a**) and Au NPs (**b**) substrates.

Moreover, the substrates present a high homogeneity, already tested with AFM, and high optical quality, probed by optical microscope images and SERS measurements. Microscopy images of the Au NS and Au NP substrates, collected with a 50× objective, are reported in Figure 11: the high density of aggregates in the NP substrate and the homogeneity of the Au NS substrate are clearly visible.

**Figure 12:**
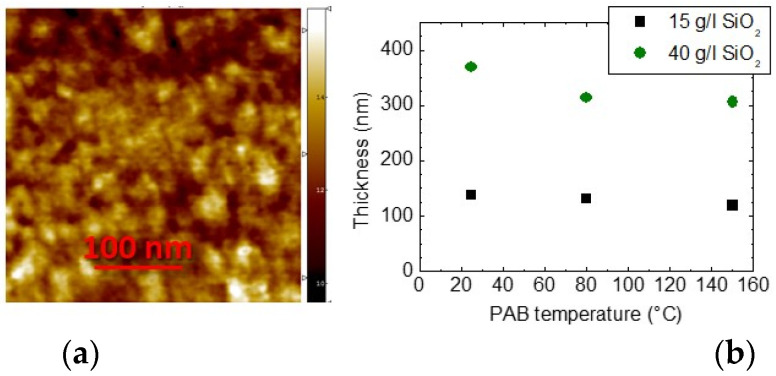
Morphological characterization of TEPS films: AFM image (**a**) and film thickness (**b**) as a function of heat treatment temperature at different sol dilutions.

The sol-gel films present a highly homogeneous pore distribution probed by AFM images and a reproducible thickness (Figure 12). 

The thickness of the sol-gel film was tested as a function of baking temperature: It can be observed that, depending on the amount of SiO_2_, higher post-application bake temperatures (PAB) can also cause a decrease in film thickness.

### 3.2. Raman Characterization of Materials and Substrates

Before the SERS sensor was realized, a basic Raman characterization was performed on the matrix and different aromatic analytes, to identify the best spectral region for xylene detection, and the determination of the plasmonic enhancement was performed on SERS substrates. 

**Figure 13:**
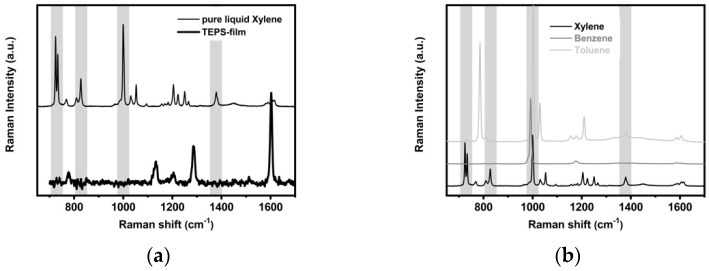
Raman spectra of the TEPS-film and of pure liquid xylene (**a**) and pure liquid xylene, toluene and benzene (**b**) in the 650–1700 cm^−1^ region.

In Figure 13a, the Raman spectra of the TEPS film and pure xylene, collected using 25 mW input power (3 spectra × 10s/spectrum), are compared. 

The Raman band positions and assignments are listed in Table 2. The grey regions show the signals of xylene (at 725–735, 810–827, 1000, and 1380 cm^−1^) that are not superimposed on the TEPS bands.

Furthermore, the comparison with the benzene and toluene spectra, collected using 25 mW input power (10 spectra × 10 s/spectrum) and reported in Figure 13b (see Supplementary Materials for Raman band positions and the corresponding assignment), confirms the choice of the previous regions as characteristic for xylene detection.

**Figure 14:**
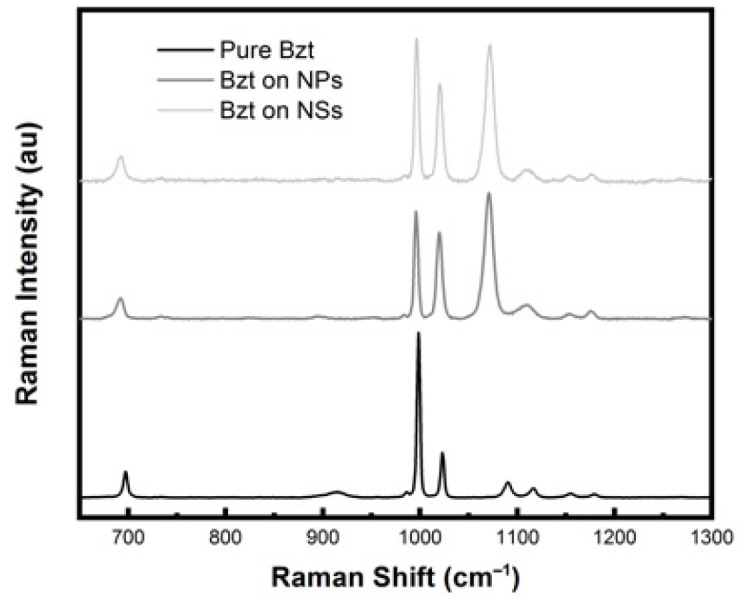
Raman spectrum of pure benzenethiol liquid and SERS spectra of benzenethiol adsorbed on NPs and NSs.

Raman signal homogeneity and sensitivity (EF) measurements were tested on NP and NS substrates, using benzenethiol as the probe molecule for the measurement of the enhancement factor [53] (see Figure 14). Optimization of NP deposition concentrations and times led to a SERS signal reproducibility on a single substrate of up to 92%, which is very good for substrates with randomly distributed NPs. The value of the enhancement factor of (1.0 ± 0.3) × 10^4^ and (3.0 ± 0.3) × 10^4^ for 633 nm excitation was evaluated, respectively, for the substrates NP and NS, by mapping the substrates at 10 random points [54] (see Supplementary Materials, Figure S1).

### 3.3. Hybrid Sol Gel Matrix Spin Coated on Plasmonic Substrate

The hybrid sol-gel matrix plays an important role in the approach of the aromatic molecules to the metallic interface and in overcoming the poor affinity in between aromatics and metals because of its porosity and the presence of bridged silsesquioxane precursors. The aromatic analyte molecules, which penetrate the porous sol-gel film, are able to interact with the phenyl bridging groups of the organic unit by π-π stacking interaction [55].

For the detection of xylene, the first idea was to directly embed gold NPs into the sol gel matrix by adding them to the precursor solution during sol gel synthesis. 

**Figure 15:**
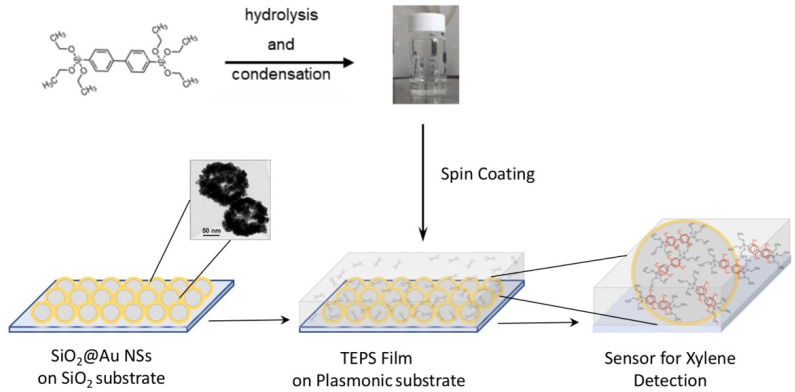
Scheme for the realization of the xylene SERS sensor.

Due to poor results, in terms of low amplification (reported in the Supplementary Materials), a different strategy was pursued: the sol-gel matrix was spin coated directly over the SERS substrate, forming the two-layer hybrid system, depicted in Figure 15. Here, the scheme of operation of the sensor is also described.

**Figure 16:**
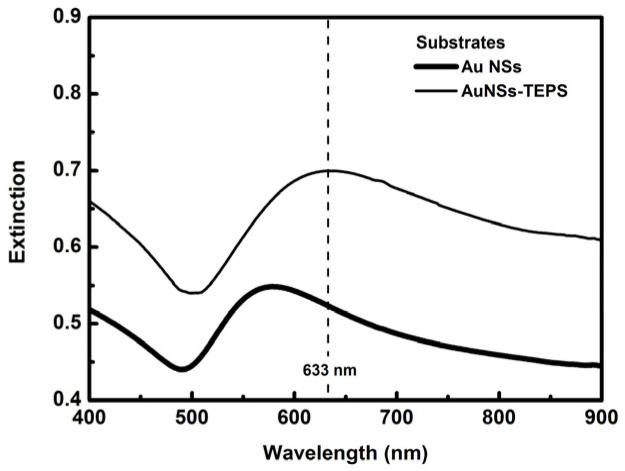
Extinction spectra of Au NSs substrate (thick black line) and AuNSs-TEPS film (thin black line) deposited on glass.

The hybrid sol was spin coated on Au NP and Au NSs (R1 = 46 nm; R2 = 67 nm) substrates to obtain films of approximately 50 nm thickness. The extinction spectra of the samples with AuNSs are reported, as an example, in Figure 16.

The extinction spectrum of the TEPS deposited on Au NSs substrates presents an increased intensity and a red shift with respect to the native Au NSs substrate. The plasmonic peak of the NSs, directly deposited on the glass substrates, is centered at 583 nm, while it is shifted at 634 nm in the presence of TEPS films. This is due to the presence of the sol gel film, which possesses a higher refractive index with respect to the air. The two sensing substrates have been demonstrated to be reproducible in terms of extinction properties. 

**Figure 17:**
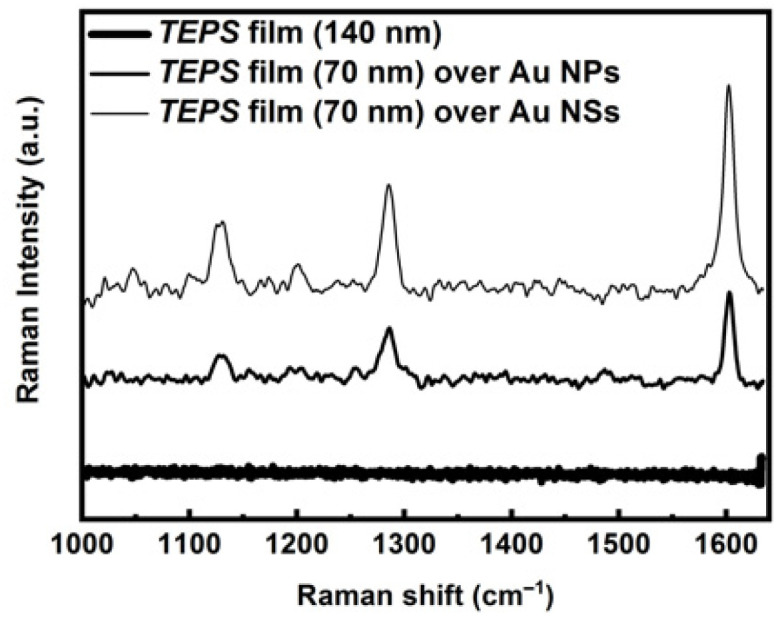
Raman and SERS spectra of TEPS film deposited on glass, Au NPs, and Au NSs substrates.

The TEPS substrates were tested using the micro-Raman setup and exciting the sample with 0.03 mW laser power; the collected Raman spectra are reported in Figure 17. 

As can be observed, the matrix signals are clearly enhanced as a result of the presence of the plasmonic substrate. A TEPS film, of about 140 nm thickness, deposited onto a glass slide, does not give any Raman signal, while, at the same laser power, thinner TEPS films of 70 nm, deposited on plasmonic substrates, show relatively strong Raman activity in the 1000–1600 cm^−1^ region. 

### 3.4. Detection of Xylene

Preliminary tests were performed incubating the Au NSs-TEPS substrates in pure liquid xylene or toluene in a closed detection setup. The collected Raman spectra (reported in Supplementary Materials) clearly show the characteristic Raman peaks of both aromatic molecules and allow the discrimination of xylene from toluene.

The enhancement efficiency of the plasmonic-sol-gel Au NSs-TEPS system was demonstrated by performing SERS measurements of substrates immersed in solutions of xylene in cyclohexane, enabling the investigation of a wide concentration range, from 0 to 800 mM. 

**Figure 18:**
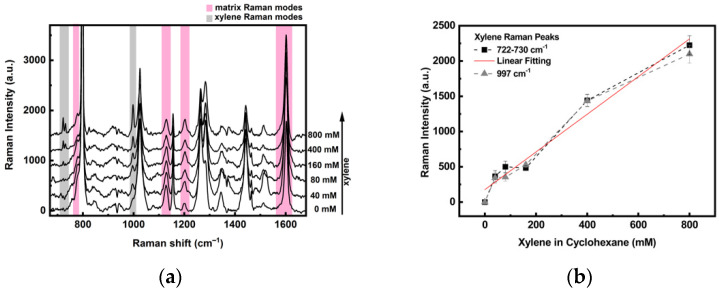
SERS spectra for the AuNSs-TEPS substrate in cyclohexane solution at different concentrations of xylene (**a**); evolution of the peak intensity of xylene as a function of concentration, with a linear fit of the signal 722–730 cm^−1^ (**b**).

In this experiment, the closed setup was used as a liquid cell: the AuNSs substrate was placed in the closed device, and the cell was filled with the organic solution and directly positioned under the SERS microscope. The collected spectra, recorded at an input laser power of 0.24 mW (20 spectra × 10 s/spectrum), are reported in Figure 18a.

SERS measurements at different concentrations of xylene in cyclohexane solution, ranging from 0 to 800 mM, are shown in Figure 18a. The signals evidenced in red are the Raman bands of the sol-gel matrix, the gray ones (at 722–730 and 997 cm^−1^) are the more intense xylene bands, not superimposed with the matrix or cyclohexane, and the others (at 798, 1024, 1155, 1264, and 1442 cm^−1^) can be attributed to cyclohexane. The xylene bands at 722–730 and 997 cm^−1^ clearly emerge when increasing amounts of the analyte are added (spectra of 40 to 800 mM) and were used to evaluate the sensitivity of the realized system to the xylene concentration. To this end, the intensity of the xylene SERS bands is reported as a function of the xylene concentration, in Figure 18b, where the linear fit of the 722–730 cm^−1^ modes is also reported and shows a good linear dependence of the signal with the xylene concentration (Adj R-square of 0.96). This value is comparable to a SERS based Napthalene sensor [56] and is within the range of literature of other SERS sensors for polycyclic aromatic hydrocarbons [57], for food products [58] and for food contaminants [59]. 

**Figure 19:**
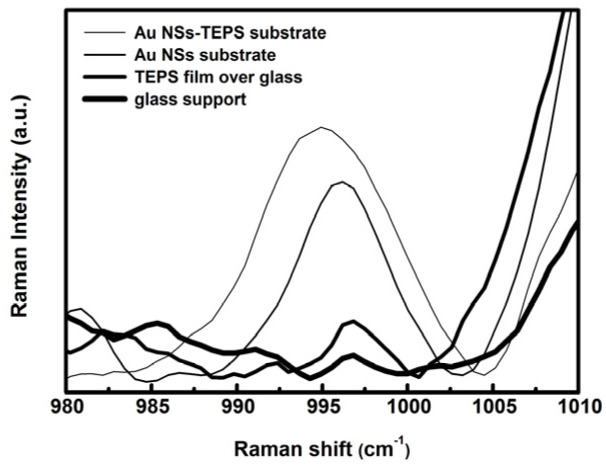
Spectral zoom in on the 995 cm^−1^ Raman band of xylene for four different substrates immersed into a 40 mM xylene solution in cyclohexane.

From SERS data, we estimate a LOD of about 40 mM xylene in cyclohexane. At this concentration, a comparison between the sensor (AuNSs-TEPS substrate), the naked plasmonic substrate (without TEPS) and a nonplasmonic substrate (TEPS film) was performed. The spectra of Figure 19 show an enhancement of the SERS signal due to the sensor design: the Au NSs substrate, without the sol-gel matrix, shows a good enhancement of the xylene Raman signal, but a further enhancement is obtained through the coupling between the plasmonic substrate and the sol-gel film. The values obtained from the integration of the Raman peak, at 995 cm^−1^, and reported in Table 3, show a signal that increases with the following ratios 1:7:14, going from the sol-gel film over a glass slide, to the NSs plasmonic film, to the coupled system.

Despite the good value of the SERS enhancement factor measured for the substrates, the LOD of xylene measured in this paper is quite high. For comparison purposes, Qian et al. measured, for toluene in solution, a LOD of 0.5 mM with the bare SERS substrate and an LOD of 0.005 mM adding a thin layer of PDMS on the substrate as a capture layer [27]; Jung et al. could separate and detect toluene and xylenes from a spiked water sample, at a concentration of around 2 mM using silver nanowires coated with a hydrophobic capture layer (LOD was not determined) [28] (see Table 4).

Relatively high LODs are expected for analytes that do not possess an affinity for the metallic surface [34]; however, in our case, the capture layer, the TEPS film, improved the detection sensitivity only to a limited extent. We attribute this limit to different possible effects:

(a) The laser heats the sample, causing desorption of the xylene molecules from the matrix. Measurement of the temperature reached by the substrate upon illumination was carried out by looking at the Stokes / anti-Stokes ratio (see Supplementary Materials), revealing that T can vary from 60 °C (laser power 8 µW) to 110 °C (laser power 800 µW).
(b) The xylene molecules absorbed in the TEPS layer lie not so close to the hot spots in the plasmonic substrate, not allowing an efficient exploitation of the SERS enhancement of the substrate.
(c) The porosity of the TEPS layer could prevent the migration of the xylene molecules toward the plasmonic amplified region at the interface with the NSs.
(d) The heating induced desorption of xylene can be overcome by using a cooling system [31] and choosing a precursor of the sol-gel film characterized by a stronger affinity for xylenes.

In conclusion, the main advantages of this plasmonic sensor are the ability to distinguish different analytes (toluene, benzene, and xylene), the possibility of tuning the chemical composition of the matrix to make it more affine to different types of analytes, and the good enhancing properties of the SERS substrate. However, the overall sensitivity of this device still needs to be improved to make it effectively applicable to the detection of BTX in the water and/or gas phase.

## 4. Conclusions

The data presented and discussed above demonstrate that the sensor system developed herein, combining a SERS substrate with a porous hybrid film, shows interesting properties in terms of easy and inexpensive realization and enhanced efficiency.

The two separate components, the Au NS substrate and the TEPS film, are easy to prepare and assemble, and their single properties work together to realize a complex system with enhanced SERS activity. The Au NS substrate provides the enhanced local field to be exploited in SERS, while the TEPS film captures the analyte near the plasmonic surface. 

Existing limitations of this system are probably due to the local heating of the sensor, which causes a fast desorption of the analyte from the diphenyl bridging group. This effect can be overcome by using a cooling system or by designing a new polysilsesquioxane system, which could show an increased interaction between the analyte and the matrix, such as a quinoide bridging group in the sol-gel system, that could provide energy transfer mechanisms. 

The potentiality of this system was demonstrated with the detection of liquid xylene, showing that the identification of xylene is specific because it can also be distinguished from benzene and toluene, because of the use of the Raman technique, which gives a fingerprint signal of the analyte. Moreover, the quantification of the xylene, at low concentrations, down to 40 mM, is obtained with a simple and easy to realize detection setup, based on the local amplification of the Raman signal, through the plasmonic resonance of NSs. The use of these properly designed plasmonic NPs allows the plasmonic resonance to be tuned, thus increasing the amplification with respect to simple gold NPs. Finally, using sol-gel porous films coupled to the plasmonic substrate, it is possible to finely and easily tune the properties of the final device.

Therefore, we can conclude that the sensor works well in terms of the specific recognition of the analyte, and therefore, it shows good potential even if it presents a high LOD. The possibility of implementing its performance is linked, on the one hand, by the use of new metal particles with greater local amplification and, on the other, by the modulation of the specific properties of the sol-gel, using new precursors with enhanced affinity toward the aromatic groups.

## Figures and Tables

**Table 1:** Radius and shell thickness values of SiO_2_@APTMS and Au NSs samples.

Sample	Radius	AFM Radius [nm]	Shell Thickness [nm]	TEM Radius [nm]
SiO2@APTMS	R1	61 ± 5	–	46 ± 3
Au NSs	R2	82 ± 7	21 ± 9	67 ± 9

**Table 2:** Raman band position and assignment of pure xylene and TEPS-film.

Raman Shift (cm^−1^)	Xylene Assignment [50,51,52]	TEPS-Film Assignment [37,38]
722–730	ν_1_	
780		ν_s_ Si-C
810–827	ν_10a_	
997	ν_18b_	
1030	ν_1a_ ν_1b_	
1050	ν_1b_	
1132		ν_s_ Si-O
1204	=C-H Out of plane ring def. vib.	
1206		=C-H Out of plane ring def. vib
1223	ν_1b_	
1250	=C-H In plane ring def. vib.	
1286		=C-H In plane ring def. vib.
1378	ν_s_ C-H (metyl)	
1602		ν_s_ C = C

**Table 3:** Peak frequency and peak area of the spectral zoom at 995 cm^−1^ for four different substrates immersed in a 40 mM xylene solution in cyclohexane.

Sample	Peak Frequency [cm^−1^]	Peak Area
Au NSs-TEPS substrate	995	1407
Au NSs substrate	996	741
TEPS film	997	107
Glass	997	33

**Table 4:** LOD of the VOC detected by SERS techniques.

Analyte	SERS Substrate	LOD [mM]	Ref.
Toluene	Au NP monolayer	0.5	xxxiv
	Au NP monolayer/PDMS	0.005	
Toluene, Xylene	Ag nanowires/hydrophobic sponge	n.d.	xxxv
Xylene	Au NP monolayer/TEPS	40	This paper

## Supplementary Materials

Available online at https://www.mdpi.com/article/10.3390/s21237912/s1.
