# Supplementary material for: Hybrid Sol-Gel Surface-Enhanced Raman Sensor for Xylene Detection in Solution
Source: Sensors (Basel). 2021 Nov 27;21(23):7912. doi: 10.3390/s21237912 (PMC8659955; doi:10.3390/s21237912)
Supplement: Supplementary file 1 [file sensors-21-07912-s001.zip › sensors-1452846-supplementary.pdf]

Supplementary Materials

Hybrid sol-gel Surface-Enhanced Raman sensor for Xylene detection in solution

Verena Weber [1], Laura Brigo [2], Giovanni Brusatin [2], Giovanni Mattei [3], Danilo Pedron [1,4], Roberto Pilot [1,4] and Raffaella Signorini [1,4,*]

[1] Department of Chemical Science, University of Padua, Via Marzolo 1, I-35131 Padova, Italy; verena.weber@unipd.it (V.W.); danilo.pedron@unipd.it (D.P.); roberto.pilot@unipd.it (R.P.)
[2] Department of Industrial Engineering, University of Padua, Via Marzolo 9, I-35131 Padova, Italy; laurabrigo@gmail.com (L.B.); giovanna.brusatin@unipd.it (G.B.)
[3] Department of Physics and Astronomy „Galileo Galilei", University of Padua, Via Marzolo 8, I-35131 Padova, Italy; giovanni.mattei@unipd.it
[4] Consorzio INSTM, Via G. Giusti 12, I-50121 Firenze, Italy;
* Correspondence: raffaella.signorini@unipd.it

1. Raman band position and assignment of Benzene, Toluene, Cyclohexane and Benzenthiol

The Raman band positions and assignments of Benzene, Toluene, Cyclohexane and Benzenthiol are listed in Table S1.

Table S1. Raman band position and assignment of pure Benzene, Toluene, Cyclohexane and Benzenthiol.

Raman shift [cm^{-1}]	Benzene [1]	Toluene [2]	Cyclohexane [1]	Benzenethiol [3]
784		1 Ring stretching		
802			Ring stretching	
992	1 Ring breathing			
998				12 Trigonal ring breath.
1002		12 Ring breath.		
1032		CH def.		
1445			CH_2 bend.	
1574				8a CC stretch.

2. Raman mapping of Plasmonic Substrates

SERS measurements have been performed on different positions of the plasmonic substrates, functionalized with benzenthiol, to evaluate their enhancement factor and homogeneity. The spectra reported in Figure S3 are collected on different positions of an AuNSs sample and show a comparable intensity, index of good homogeneity of the samples. The intensity of the Raman mode at 998 cm^{-1} has been used to calculate the enhancement factor of the substrates [4,5].

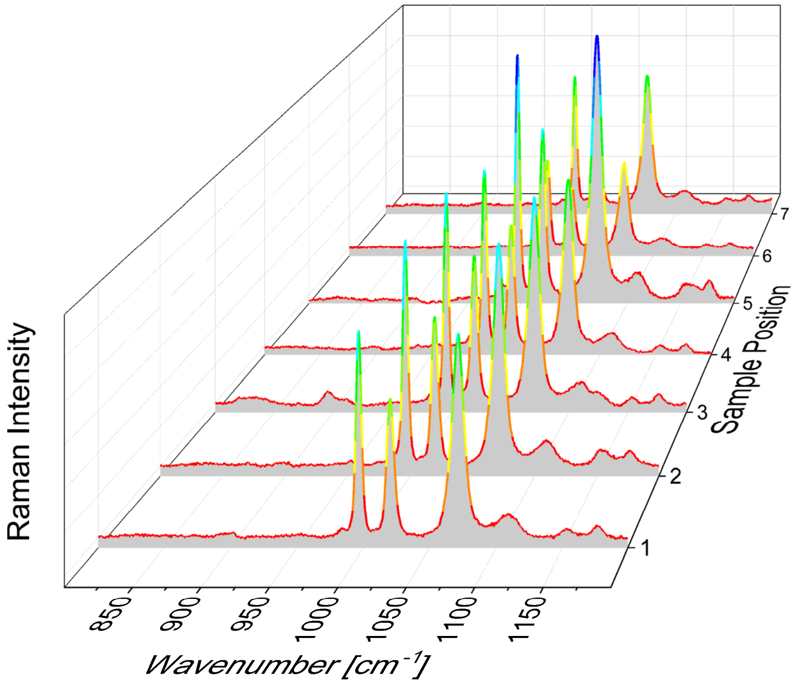

Figure S1. Raman spectra of benzenthiol on plasmonic substrate.

3. Experimental setup detection and first test

A homemade closed detection setup was realized, which allows one to incubate the plasmonic sample with the Xylene analyte and to directly perform the detection measurements in the closed cell through an optical window. The picture of the detection device is shown in Figure S1. It consists of a round teflon support with inlet and outlet tubes where a plasmonic sample of dimension 2.6 x 1.5 cm could be located. The device can be closed from the top through a teflon ring with a polymer o-ring resting over a thin glass window. The device can be hermetically closed through small screws. SERS measurements are performed by placing the device directly under the microscope objective, which should have a working distance corresponding at least to the thickness of the optical glass window.

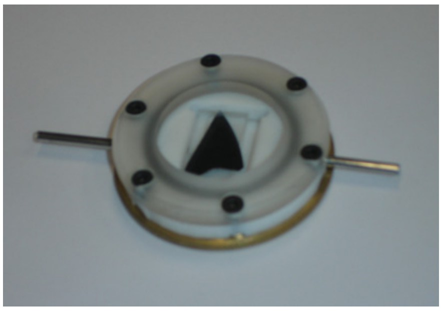

Figure S2. Picture of the closed homemade SERS detection setup.

First, tests were done incubating the Au NSs-TEPS substrate in pure liquid Xylene; the substrates were air-dried and then put into the closed detection setup. The results with 785 nm excitation wavelength and 0.8 mW laser power (50x objective and 20x30 s spectra accumulation) are shown in figure S2, where the signal of Xylene on the Au NSs-TEPS substrate is also compared with a sample incubated with toluene on a similar substrate. The Raman spectra are also collected as soon as the sample is inserted into the detection seup and after a 10 and 30 minutes later.

The xylene and toluene signals on Au NSs-TEPS substrates are compared with pure liquid xylene and toluene signals: the characteristic Raman peaks are clearly visible right after incubation in the pure liquid and allow discriminating Xylene from toluene. After only ten minutes, the signals of Xylene are significantly lower, indicating a fast desorption of the analyte molecules from the sol-gel matrix, while after 30 minutes, the signals of toluene are lower but still detectable.

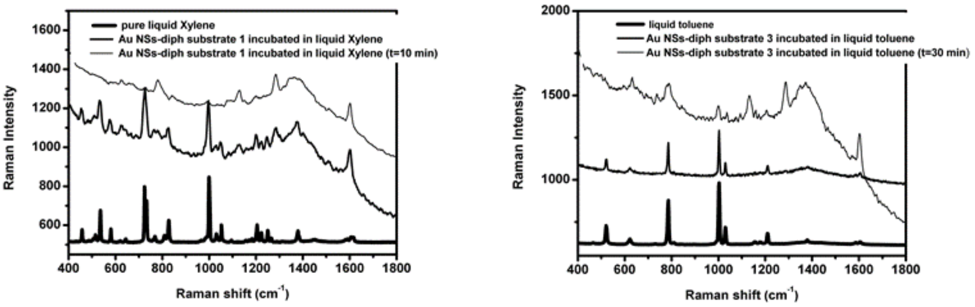

(**a**) (**b**)

Figure S3. Raman/SERS spectra for AuNSs-TEPS substrates incubated in liquid Xylene (**a**) and toluene (**b**).

4. Local Temperature detection

Local temperature was measured using Stokes and Anti-Stokes Raman measurements performed on a 'naked' SERS substrate functionalized with benzenethiol. By recording both the Anti-Stokes and Stokes signals of a given Raman mode, it is possible to calculate the temperature on the substrate through the relation [6]:

$$\frac{I_{aSto}}{I_{Sto}} = \left(\frac{\omega_L - \omega_i}{\omega_L + \omega_i}\right)^4 e^{-\frac{\hbar\omega_i}{k_B T}}$$

For this measurement, a calibration with a liquid reference is needed, in order to perform a relative temperature measurement, independent of instrumental factors. In this case, liquid toluene was measured and the $k_B T$ value was set at room temperature. In doing so, an instrumental correction factor c could be calculated, which can be then applied to the measurements on the SERS substrates. Measurements were made at three different incident laser powers, ranging from 80 μW to 0.8 mW. The temperatures on the substrate are found to range from 60 to 110 °C, depending on the laser power used.

5. References

1. Dollish, F. R.; Fateley, W. G.; Bentley, F. F. *Characteristic Raman frequencies of organic compounds*; John Wiley & Sons, Inc: New York, USA, **1974**.
2. Hitchcock, A. P.; Laposa, J. D. Vibrational Frequencies of Toluene-d5. *J. Mol. Spectrosc.* **1975**, 54, 223-230, doi.org/10.1016/0022-2852(75)90076-4.
3. Joo, T.H.; Kim, M.S.; Kim, K. Surface-enhanced Raman scattering of benzenethiol in silver Sol, J. *Raman Spectrosc.* **1987**, 18, 57–60, doi: 10.1002/jrs.1250221214.
4. Le Ru, E.C.; Blackie, E.; Meyer, M.; Etchegoin, P.G. Surface Enhanced Raman Scattering Enhancement Factors: A Comprehensive Study. *J. Phys. Chem. C* **2007**, 111, 37, 13794–13803, doi: 10.1021/jp0687908.
5. McFarland, A.D.; Young, M.A.; Dieringer, J.A.; Van Duyne, R.P. Wavelength-scanned surface-enhanced Raman excitation spectroscopy. *J. Phys. Chem. B* **2005**, 109, 11279–85, doi:10.1021/jp050508u.
6. Kip, Bert J.; Meier Robert J. Determination of the Local Temperature at a Sample during Raman Experiments Using Stokes and Anti-Stokes Raman Bands. *Appl. Spectr.*, **1990**,44 (4), 707-711.
